# Regression analyses of event-related brain potentials dissociate general reading experience and domain-specific knowledge sources during language comprehension

**DOI:** 10.3389/fcogn.2026.1856123

**Published:** 2026-08-24

**Authors:** Melissa Troyer

**Affiliations:** Department of Psychology, University of Nevada, Las Vegas, Las Vegas, NV, United States

**Keywords:** domain knowledge, ERPs, language comprehension, multimodal processing, N400, regression ERPs

## Abstract

How do different sources of knowledge and experience shape how we make meaning from language in real time? The present work capitalizes on recent advances in ERP analysis (the regression ERP, or rERP approach, combined with AIC model comparison to compare how various measures of knowledge and experience related to millisecond-by-millisecond ERP time-course data recorded as individuals read sentences about a fictional world (Harry Potter; HP) one word at a time. Participants were individuals who ranged in the experiences they had contributing to their HP knowledge, including the number of times they had read each book and/or seen each movie. These participants also completed an objective measure of domain knowledge using a short trivia quiz, as well as more general measures of reading experience. HP-based measures out-performed general reading experience for multiple indices of word understanding. Notably, self-reported experience with HP movie-watching best explained N400 “related anomaly” effects, which reflect the immediate availability of linguistically inappropriate but event-related words. Thus, the rich multimodal experiences of watching movies may, at least in some cases, accelerate knowledge use during language understanding compared to purely linguistic experiences of reading. These findings run counter to theories on which meaning is based on linguistic experience alone, implicating multimodal contributions. More generally, these analyses illustrate how exploratory model set comparisons and continuous variable rERPs yield new insights into the time course of information processing during reading.

## Introduction

When we read or listen to a language we know, we immediately bring to mind a host of information that is related to each word, sentence, and situation described. This information includes information about words that are similar in sound or written form (Grainger et al., 1989; Laszlo and Federmeier, 2009), words that are meaningfully related and/or frequently co-occur (Balota, 1983; Federmeier and Kutas, 1999), objects and entities that are inferred from the sentence context, even if never explicitly mentioned (McRae and Matsuki, 2009), and more broad, general knowledge about the events being described in the sentences (Metusalem et al., 2012), including perceptual information (Amsel et al., 2015). This information comes from our individual stores of knowledge, accrued over a lifetime of experience, which vary from person to person and can have immediate impact on perception and cognition (cf. the literature on expertise; Ericsson et al., 2018). It is well attested that differences in print exposure (for example, measured using tests of author and magazine title recognition; Stanovich and West, 1989; Acheson et al., 2008) impact both real-time processing (e.g., Ng et al., 2017; Huettig and Pickering, 2019) and relative engagement of differing neural systems (Song et al., 2026) underlying language comprehension. Yet little is known about how specific sources of experience combine to shape how we make meaning from language, as it happens. The present study leverages recent advances in EEG/ERP methods to directly compare the impact of different sources of knowledge on real-time access to word meaning.

How we represent meaning is a topic of much debate within cognitive science. On one extreme, distributed semantics hypotheses propose that people can represent word meanings solely based on their co-occurrence with other words (“You shall know a word by the company it keeps”; Firth, 1957; reviewed in Lenci, 2018). Recent advances in the use and application of large language models lend some credence to this claim, suggesting that given enough language input, these models can “understand” and produce linguistic content that is grammatical, plausible, and pragmatically appropriate. However, few would argue that linguistic experience is the only source of knowledge we use when comprehending language (e.g., Andrews et al., 2009), and indeed, some pragmatic inferences are difficult for large language models, since they do not “know” about real-world experiences and related object affordances (e.g., Jones et al., 2022, 2024). Opposing theories highlight the importance of multimodal, grounded cognition, arguing that language understanding depends (to some extent) on embodied experiences in the real world (e.g., Barsalou, 1999). These theories of embodied/grounded cognition argue that we continue to use mental traces of these experiences as we understand language, including the use of neural and cognitive systems that are involved in the actual perceiving of sensory input and in the planning and execution of actual motion (Barsalou et al., 2003). This view is supported by findings that word meanings seem to be distributed across the cortex (Huth et al., 2016), with non-arbitrary correspondences between word meanings and sensory-motor neural systems (e.g., words having to do with leg-related actions, like kicking, recruit parts of the motor system that are involved in planning leg/foot motion; Hauk et al., 2004).

In addition to spoken language and multi-modal experiences grounded in the real world, fictional input (both linguistic and otherwise) constitutes an additional source of knowledge that contributes to how we understand the world (Marsh et al., 2003). Over the past decade, my collaborators and I have explored a fictional world in depth in order to ask questions about how specific domain knowledge impacts language comprehension (Troyer and Kutas, 2018, 2020; Troyer and Federmeier, 2026; Troyer et al., 2019, 2022, 2024, 2025, 2026). This research, focusing on the narrative world of Harry Potter, has examined how naturally-occurring variation in knowledge impacts how people make meaning from language in real time. We had initially assumed that much of this specific knowledge would rely on individuals' experience with reading the HP books; however, our studies have shown that there is also substantial variability in how many times participants had experienced HP in other modalities, including watching movies and participating in real-world events like attending conventions and going to amusement parks. These self-report measures tend to correlate highly with each other, with objective scores on HP trivia quizzes, and with more general measures of reading experience, making it difficult to sort out which type of knowledge/experience might best capture experimental effects using traditional analyses.

The present work leverages recent advances in regression ERP analyses and exploratory model comparison approaches to address this question head-on, directly comparing regression models fit to the same data to ask which measure(s) best capture effects of interest. The next sections briefly overview relevant background describing an ERP measure of semantic access (N400 amplitudes) followed by the rERP/model comparison approach used in the present analyses.

### ERP studies of language and knowledge

Since the original report by Kutas and Hillyard in 1980, N400 brain potentials have proven to be highly useful tools for research on how people make meaning from language (Kutas and Federmeier, 2011). N400 brain potentials are so called because they are negative in polarity (relative to a bimastoid reference) and peak approximately 400 ms after potentially meaningful stimuli—including words as well as pictures, line drawings, and “pseudowords” (Kutas and Federmeier, 2000; Laszlo and Federmeier, 2009). Over the years, different theoretical accounts have proposed that N400s reflect (post-lexical) semantic integration processes (e.g., Brown et al., 2000), lexico-semantic retrieval processes (e.g., Brouwer et al., 2012), and processes indexing semantic “error” (i.e., the difference in meaning between a predicted word and the actual input) in predictive processing frameworks (e.g., Rabovsky et al., 2018). Regardless of which theory comes closest to describing N400s, all of them share the consensus that N400s reflect some aspect(s) of meaning processing. They are small in amplitude when processing is facilitated by various aspects of context—including sentential context (Kutas and Hillyard, 1980, 1984), the meaning of a predictable (but never presented) word (Federmeier and Kutas, 1999), word frequency (Van Petten and Kutas, 1990), and who the speaker is (Van Berkum et al., 2008)—and large when an incoming word is less facilitated by context, meaning that more information has to be made active to process the meaning of the word (reviewed in detail in Federmeier, 2022).

Importantly for the present study, N400 potentials are also highly sensitive to the comprehender's specific knowledge. In an initial study, Troyer and Kutas (2018) used a specific domain of knowledge, the narrative world of Harry Potter (HP), so that they could measure naturally-occurring individual differences in knowledge of that domain, which was quite popular at that time. They asked undergraduate participants, many of whom had grown up with the series and were quite knowledgeable, to read sentence pairs describing fictional “facts” about the series. Half of these ended with the correct/contextually-supported word (e.g., “Harry has a Patronus. It takes the form of a stag.”) and half ended with an incorrect word that was designed to seem generally plausible (e.g., “Harry has a Patronus. It takes the form of a lion.”)—except for the initiated HP fans who had the requisite experience to know better. As expected, the authors observed the canonical effect of sentence context on N400 amplitudes to words in HP sentences, with large N400 amplitudes to the incorrect/unsupported words and reduced N400 amplitudes to words which were correct/supported by the context. Notably, the size of this effect (that is, the difference in mean N400 amplitude to unsupported minus supported words) was positively correlated with participants' knowledge of the HP domain, measured objectively using a short trivia quiz. In other words, high-knowledge individuals showed rapid effects of context on semantic processing while there was no observable effect for individuals with the least knowledge (see Figure 4 in Troyer and Kutas, 2018). These results showed, for the first time, that participant-level individual differences in knowledge (even knowledge gleaned from fiction) can have an immediate impact on how people make meaning of words in context.

A subsequent study used N400 potentials to probe the nature of the knowledge activated by the context. Troyer and Kutas (2020) constructed a new set of sentences describing 156 fictional facts about the world of HP. This time, the sentences could end in three ways: with the contextually supported word (as before), a contextually unsupported and anomalous word, or a word which was contextually unsupported/incorrect but *related* in some way to the events described by the sentence context and/or the correct ending (see Table 1). Consistent with previous studies using similar “related-anomaly” paradigms (e.g., Federmeier and Kutas, 1999; Metusalem et al., 2012), N400 amplitudes were largest for the unrelated/anomalous words, smallest for the correct/supported words, and intermediate for the unsupported but contextually-related words. The authors also found that the size of both the contextual support effect (mean N400 amplitudes to unsupported/unrelated minus supported words) *and* the related anomaly effect (mean N400 amplitudes to unsupported/unrelated minus unsupported/related words) were significantly correlated with participant-level HP knowledge. These findings strongly suggest that individuals make quick use of their knowledge in a deep, rich way during sentence comprehension, drawing on their experience to bring to mind aspects of the people, places, and things involved in events and themes from fictional books and movies.

**Table 1:** Sample sentence materials by condition.

Sentence frame	Supported	Unsupported-related	Unsupported-unrelated
There is one main bank in the wizarding world. It is run by	goblins	werewolves	Alohomora
Sybill Trelawney is a Hogwarts professor. She teaches	Divination	Transfiguration	Basilisk
There is a branch of magic focused on changing the form of objects. It is called	Transfiguration	Divination	Alley
Ginny opens the Chamber of Secrets and unleashes and ancient monster. It turns out to be a	Basilisk	Diary	Shunpike
Professor McGonagall recruits Harry for the Gryffindor Quidditch team. She saw him save Neville's	remembrall	broomstick	dog
Before Harry's second year, he is rescued by Ron, Fred, and George Weasley. They pick him up in a flying	car	Dursleys	Draco

**Figure 1:**
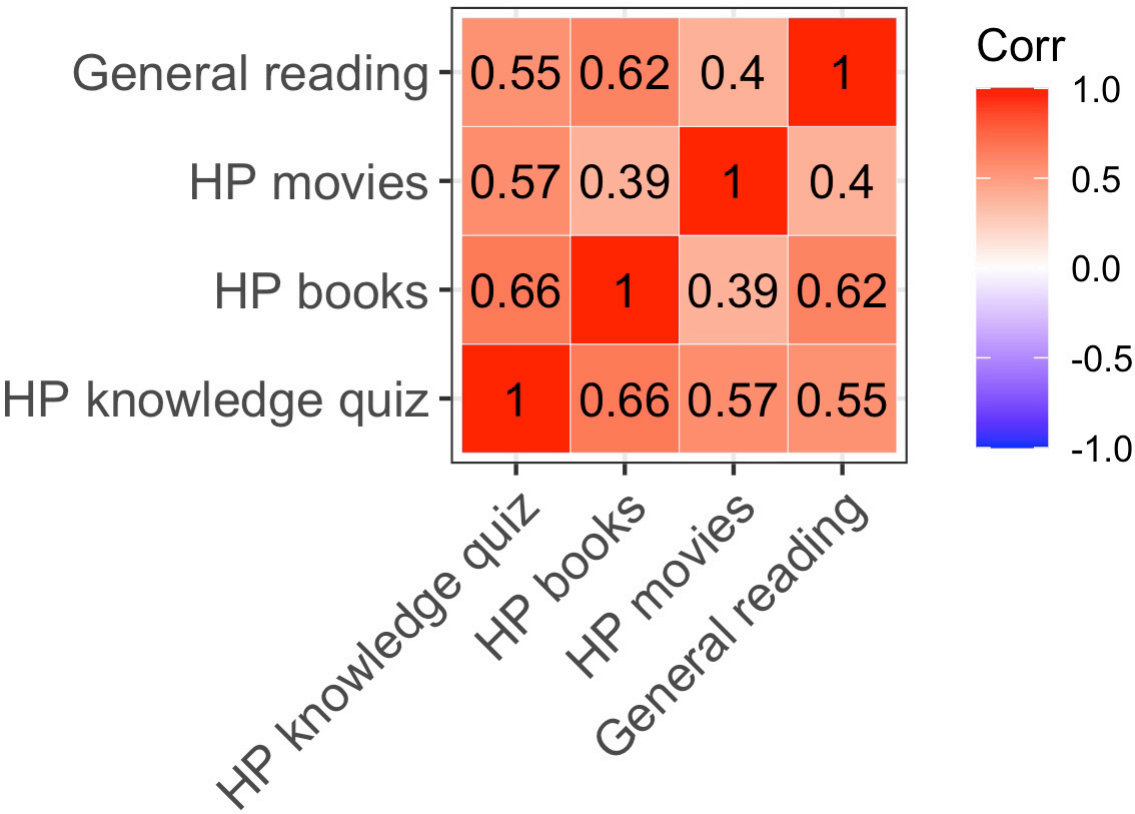
Intercorrelations among measures of individual differences. All values are significant at α = 0.01.

In the Troyer and Kutas (2020) study, the authors focused primarily on a single measure of individual differences in HP knowledge—their trivia quiz measure of HP, which tested individuals using 10 multiple-choice questions. However, they also collected additional individual difference measures, including a measure of general reading experience (based on self-report) as well as a self-report HP experience questionnaire in which participants indicated how many times they had read each book and seen each movie. Unsurprisingly, all four measures (HP knowledge/trivia quiz scores, HP book experience, HP movie experience, and general reading experience) were moderately-to-strongly inter-correlated (summarized in Table 2 and Figure 1), making it difficult (using standard regression modeling approaches) to parcel out which measure(s) contributed most strongly to the observed N400 effects of contextual support and related anomaly. The next section describes a recent advance in ERP analysis methodology which allows for a direct comparison of different regression models fit to the same dataset. This advance allows us to re-analyze the existing Troyer and Kutas (2020) data in order to ask which types of experience—reading books (generally), reading HP books (specifically), or watching HP movies—best explain the data.

**Table 2:** Individual differences.

Measure	All participants			Relatively high HP			Relatively low HP		
	Mean	SD	Range	Mean	SD	Range	Mean	SD	Range
HP quiz	6.06	(2.50)	[1, 10]	8.48	(1.25)	[7, 10]	3.77	(1.07)	[1, 5]
HP books	7.75	(8.82)	[0, 28]	13.00	(10.74)	[0, 28]	3.14	(3.30)	[0, 10]
HP movies	18.02	(8.95)	[0, 28]	23.76	(5.37)	[11, 28]	12.82	(9.25)	[10, 22]
General reading experience	−0.04	(0.70)	[−1.19, 1.69]	0.36	(0.76)	[−1.18, 1.69]	−0.37	(0.43)	[−1.19, 0.37]

### Regression ERP approach

In psycholinguistics, many researchers use linear mixed effects models to capture random effects of both participants and items. There are different approaches to how researchers make statistical inferences from these models. Perhaps the most common is to directly interpret beta coefficients for fixed effects using the lmerTest library's estimation of *p*-values (Kuznetsova et al., 2017). However, this approach may not always be ideal because appropriate interpretation depends on how the models are specified (e.g., are the data centered and scaled? Are the predictors sum-coded or treatment coded?, etc.). A more powerful approach for inferential statistics using regression modeling is to conduct nested model comparison, in which two models, fit to the same dataset, one containing a subset of the other's predictors, are compared using a log-likelihood ratio or Chi-squared test (e.g., using lmerTest's anova() function), which takes into consideration not only which model best fits the data but also the number of free parameters (alternate approaches would be to use other goodness-of-fit measures; see discussion in Bates et al., 2015b). In these approaches, the “best” or “preferred” model is the one that best explains the data after correcting for the number of predictors. Inferential statistics about individual predictors can thus be made by computing two models, one of which drops the specific term of interest (reviewed in Winter, 2019). For example, when modeling N400 amplitudes as a function of HP knowledge and condition, one might wish to know whether the effect of interest (e.g., contextually supported word vs. unrelated/anomalous word) and the individual difference measure of HP knowledge interact. One could thus compare the following two nested models:


$$\begin{array}{l} \text{m}1:\;\text{N400}\;\sim \;1+\text{HP\_Knowledge}+\text{Condition}\\ \;+\;\text{HP\_Knowledge X Condition}\\ \text{m}2:\;\text{N400}\;\sim \;1+\text{HP\_Knowledge + Condition} \end{array}$$

If the first model is preferred, the inference can be made that there is a significant interaction of HP knowledge and condition.

Many ERP studies of language use the types of linear mixed-effects models described above to make statistical inferences about mean voltage amplitudes in pre-defined time windows and spatial regions of interest (ROIs). However, the visualization of ERP data does not reflect the construction of these models. It is the convention to plot ERPs amplitudes (averaged across all frequency bands) over time, such that the viewer can understand the component of interest (e.g., an N400, or a P300, or an N1, etc.) in the context of the electrical activity immediately preceding it (including a baseline period prior to event onset) and succeeding it. These grand-averaged ERPs are most typically created by computing the point-by-point average for each participant and channel, then point-by-point averaging the subject-averaged data to create a grand average; they can also be computed as the point-by-point average across all trials, pooled (without first averaging by subject).

Thus, ERPs are typically analyzed and visualized using different approaches. Recently, Smith and Kutas (2015a,b) described an approach which allows research to more directly inspect individual predictor-level effects in regression models across a time series. As Smith and Kutas (2015a) describe, averaging can also be modeled as a simple regression. They present a novel framework for modeling ERP timeseries data in which, instead of a simple average for each data point, a regression model is used to estimate that data point (so called regression ERPs, or rERPs). The resulting predicted values can be plotted, much like a traditional subject- or grand-average; and, in addition, the values of individual predictors (i.e., beta-coefficients) can also be plotted over time. This approach is especially powerful because it allows researchers to visualize (and perform inferential statistics on) not only categorical effects (e.g., the effect of an experimentally manipulated condition), but also continuous effects (e.g., the effect of a measured individual difference or item-level variable) *and* any desired interactions of any combination of categorical and continuous effects. In addition, any sort of regression model can be used—so it is possible to apply more complex regression models that use a combination of fixed and random effects (i.e., linear mixed effects models; Bates et al., 2015a) to create rERPs (see examples of this application in Aurnhammer et al., 2021, 2023; Delogu et al., 2025).

One application of rERPs is specifically for data visualization. My colleagues and I have used this approach to visualize predicted ERPs (analogous to grand-average ERPs) modeled from trial-level data including both continuous and categorical predictors (e.g., Troyer et al., 2019, 2026). In these studies, statistical inferences were made over predefined time windows and spatial ROIs (e.g., N400s were analyzed over a centroparietal set of electrodes in the 250-500 ms time window). However, the rERP framework provided a way to visualize ERPs for *hypothetical* values that might or might not have ever been observed. For example, Troyer et al. (2019, 2026) modeled HP knowledge as a continuous (z-scored) variable. They could thus enter a hypothetical value of ±1 or 2 standard deviations from the mean as the value of HP knowledge and examine the ERPs that would be predicted for various levels of other variables; in that case, two categorical predictors: (1) whether or not a participant reported having known a given HP fact and (2) whether the fact was relatively well-known or less-well-known across a group of norming participants. Thus, a major benefit of the rERP approach is its flexibility in data visualization, which also better captures the statistical model used to make the ultimate statistical inferences. However, in the application described above, there is still a disconnect between the visualization (in which an estimate is plotted at every time point sampled) and the statistical models used for inference, since the statistics are computed on average amplitudes in a specific time/spatial ROI. In other words, rERPs can be used to visualize estimated ERP timeseries but need not reflect actual inferential statistical output; this is analogous to the standard approach of computing statistical analyses on subject-averaged mean amplitudes in predescribed time windows while visualizing ERP timeseries across an entire epoch. This is not necessarily a problem. Given that many ERP components, including N400s, are already well-characterized in terms of spatial and temporal ROIs, it is not necessary (or advisable) to compute statistical analyses at every time point and electrode site. Such an approach intensifies computational complexity and requires correction for multiple comparisons (for an ERP study using a modest 26 channel electrode array and a 250 Hz sampling rate, a study with a second-long epoch would thus yield 6,500 statistical tests). However, in exploratory data analysis, rERPs offer a powerful tool for identifying new or unexpected patterns.

The present study combines the use of rERPs with an exploratory model comparison approach that is not often used in psycholinguistics, namely, AIC_min_ Δ. This approach uses goodness-of-fit metrics (here, Akaike Information Criteria; AIC, reviewed in Burnham and Anderson, 2004) to compare how different regression models, fit to the same dataset, perform *relative to one another* in explaining the data. An advantage of this approach is that the models need not be nested. This approach is thus less tied to exploring potential hypotheses and most well-suited for exploratory questions about which measure(s) are the best/most meaningful predictors of the observed data. This approach is often used in fields like behavioral ecology, where regression models with many different variables (e.g., time of day, location, etc.) are used to explore novel patterns in observational data (Burnham et al., 2011) and has recently been used for exploratory data analyses in EEG studies of language (Urbach et al., 2020).

To assess AIC_min_ Δ, AIC is first computed for each model considered, and the best-fitting model is identified—i.e., the one with the lowest AIC value, or AIC_min_. This AIC_min_ value is then compared to that of each of the models under consideration. AIC_min_ Δ is defined as the difference between the AIC of the best-fitting model (AIC_min_) and that of each model. Thus, by definition, the model with the lowest AIC has an AIC_min_ Δ of 0. Burnham and Anderson (2004) provide guidelines for how to interpret the resulting statistics: “…models having Δ_i_ ≤ 2 have substantial support (evidence), those in which 4 ≤ Δ_i_ ≤ 7 have considerably less support, and models having Δ_i_ > 10 have essentially no support” (pp. 270–271; see Urbach et al., 2020, for further discussion in applying this method to ERP data).

This approach affords the ability to ask a question that had been unanswerable using standard ERP analyses; namely, what measure of individual differences (out of four considered here) best explains N400 effects of contextual support and related anomaly? In other words, what types of experience provide the most explanatory power in terms of how people make sense of language, in real time?

## Method

### Dataset

The present dataset comes from Troyer and Kutas (2020). This study had originally included 40 sentence pairs describing general topics (control sentences) and 156 sentence pairs describing fictional “facts” about the narrative world of HP. For control sentences, each sentence pair ended either in the best completion or another word that was unexpected but designed to seem plausible. For the HP sentences, sentences ended either in the appropriate/correct word, an incorrect (and often completely anomalous) but contextually related word, or an unrelated anomalous word. In the present analysis, only the 156 HP sentences of interest were analyzed (see examples in Table 1).

During the study, the electroencephalogram (EEG) was recorded as participants read sentence pairs. Participants were instructed to read these sentences for meaning and that they would be asked to answer questions about the sentences after the experiment. For all sentence pairs, the first sentence was presented all at once. Participants pressed a button to move on to the second sentence, presented at an SOA of 500 ms (200 ms on, 300 ms off). ERPs were analyzed to the critical words, which were always sentence-final. During setup for the ERP study, participants completed the author and magazine recognition tasks (described below). After the ERP portion of the study, participants completed an additional series of behavioral tasks. The first task asked participants to read the same sentence contexts again and provide the word they thought would best complete the sentences (regardless of what type of word they had actually read in the ERP study). These behavioral data were not analyzed in the present analysis / study. Next, participants completed several measures of individual differences (see below). See Troyer and Kutas (2020) for more methodological details.

### Measures of individual differences

Four measures of individual differences from Troyer and Kutas (2020) are detailed below. The Author and Magazine Recognition tests and Media and Reading Habits Questionnaire were completed as participants underwent set-up for the EEG study; all other measures were collected after the EEG study.

### Harry Potter knowledge quiz

An objective estimate of participants' knowledge of Harry Potter was computed from their score on a trivia-style quiz containing ten multiple choice questions (see Troyer and Kutas, 2018, for more details).

### Self-reports of Harry Potter book and movie experience

Subjective scores of participants' experience with the Harry Potter universe were measured via a comprehensive questionnaire which asked about multiple ways they had engaged with Harry Potter (details in Troyer and Kutas, 2020). The present analyses include two estimates from this measure: self-report of how many times participants (1) had read the HP books and (2) had seen the HP movies.

### Measures of print exposure and reading experience

To estimate individual differences in print exposure, participants completed author and magazine recognition tests (ART and MRT; Acheson et al., 2008). These asked participants to view checklists of 80 names (ART) or titles (MRT) and choose the ones they recognized as authors or magazines. Scores were calculated by subtracting the proportion of false alarms from the proportion of correct names checked. Participants additionally completed a media and reading habits questionnaire modeled on Stanovich and West (1989); see details in Troyer and Kutas (2018). This questionnaire contained a measure of reading experience that is known to be predictive of a number of language-related outcomes, namely, the number of names an individual produced when asked to list their favorite authors. For a composite measure of reading experience, four z-scored measures from the following tasks were averaged: ART scores, MRT scores, MRH scores, and number of authors produced (as in Troyer and Kutas, 2020).

### Analysis

The EEG processing pipeline is briefly described before an explanation of the current regression ERP approach. More details are available in Troyer and Kutas (2020).

### EEG epoching and artifact correction

Single-trial epochs of EEG data were extracted from the continuous recordings (which used an analog bandpass filter from .01 to 100 Hz and were sampled at 250 Hz) from 200 ms prior to the onset of a critical word until 900 ms post-critical word. These were subsequently averaged by participant for each condition. For each electrode, the 200 ms pre-stimulus baseline was subtracted from the waveform. Each trial was visually inspected for artifacts, and trials contaminated by eye movements, blinks, muscle activity, blocking, or other artifact were removed from subsequent analysis using a script that included tailored thresholds (based on visual inspection) for each participant in ERPlab (Lopez-Calderon and Luck, 2014). This led to an exclusion of 15% of trials in each condition (Supported, Unsupported-Related, and Unsupported-Unrelated).

### Regression ERP visualization and analysis

To compute rERPs, difference waves were computed between condition pairs (contextual support effect: Unsupported-Unrelated minus Supported; related anomaly effect: Unsupported-Unrelated minus Unsupported-Related) for each participant. These difference waves were modeled, timepoint by timepoint, as a function of predictors of interest. Four separate linear regression models were fit, each incorporating an intercept term and a single fixed effect corresponding to an individual-difference variable: HP knowledge quiz score, HP book reading experience, HP movie watching experience, and general reading experience.^1^ For each measure, two separate models were computed, one for each dependent variable (effect of contextual support, effect of related anomaly). Because each of the individual differences effects was normalized and centered (z-scored), the intercept terms always represent the mean difference score (across participants) and are thus the same across models. Plots were created to visualize the intercept and the predicted effect of the individual difference variable examined.

### AIC model comparison and statistical inference

For statistical inferences in this exploratory framework, the AIC_min_ Δ metric was used to directly compare the goodness-of-fit of considered models (four models compared in each analysis). For each time point, the minimum AIC value is computed as the AIC value of the best-fitting model. Thus, the difference between this value and the AIC value of the best-fitting model is defined as 0 (i.e., AIC_min_ Δ = 0). The difference between the AIC_min_ value is also compared against the AIC value of each of the other models, resulting in a AIC_min_ Δ metric for each of the four models. Larger AIC_min_ Δ values indicate poorer fit relative to the best model (of those considered). This approach is therefore useful for determining which model is the best out of a set of defined models, but does not provide information about overall explanatory power of the best model—it has nothing to say about the amount of variance explained. Given that experimental psycholinguistic studies such as this one aim to detect effects (given carefully controlled experimental manipulations), rather than explain variance, this is a powerful approach for comparing *relative* explanatory power of different variables in experimental contexts.

Data are visualized in raster plots, with darkness of color indicating the relative explanatory power of each model considered, across four representative midline electrodes (midline prefrontal (MiPf); midline central (MiCe); midline parietal (MiPa); and midline occipital (MiOc)). Lower values (here, visualized in the lightest color) indicate the best fit. The darkest colors indicate the poorest fit.

**Figure 2:**
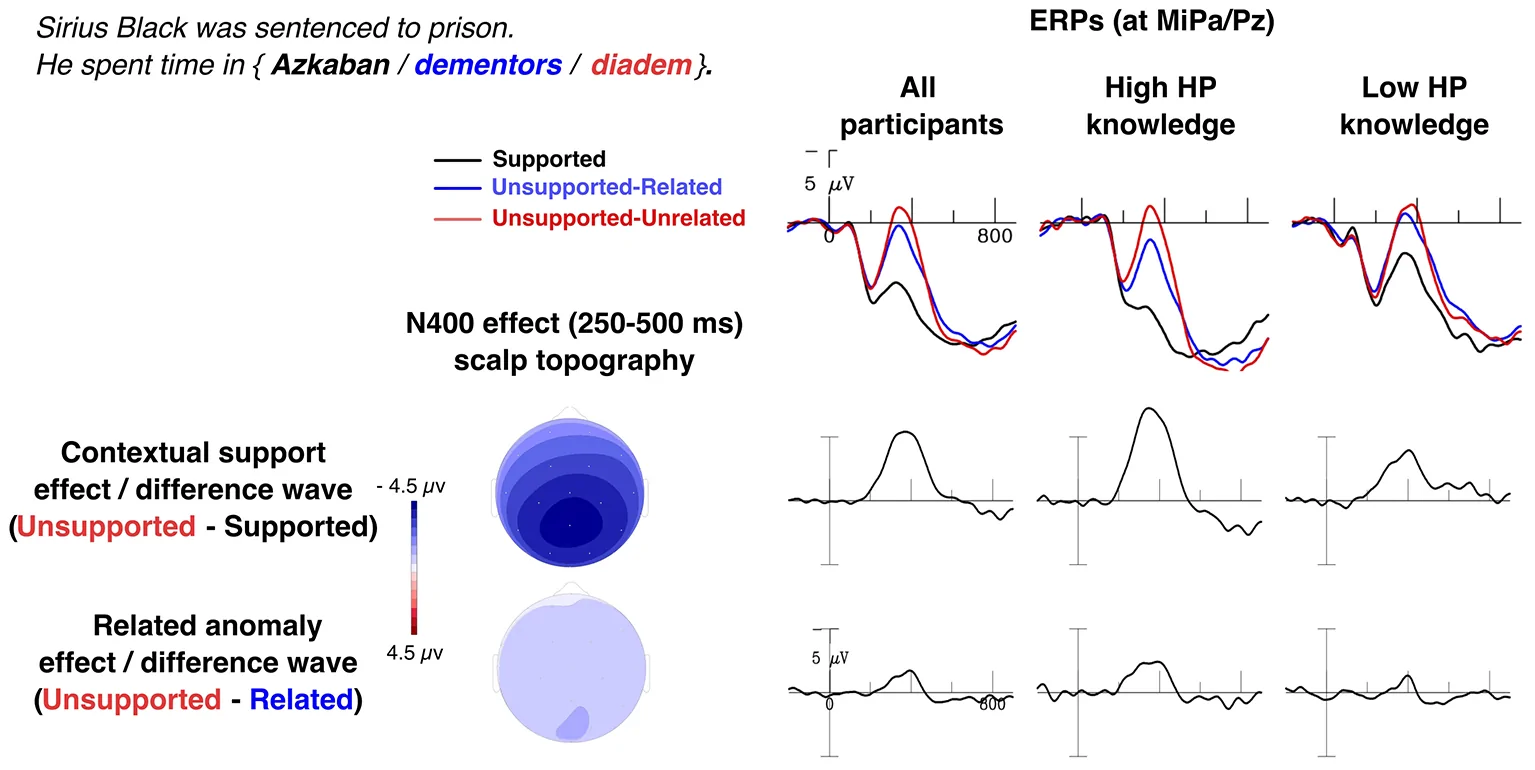
N400 effects between 250–500 ms had a broad distribution and centro-parietal maximum, as illustrated in the topographical scalp plots on the bottom left. To the right, ERPs are plotted at a representative electrode (MiPa/Pz) for all participants, relatively high-HP-knowledge participants, and relatively low-HP-knowledge participants, for each condition **(top)**. Contextual support and related anomaly effects are also plotted at MiPa/Pz for all the same groups **(bottom)**.

Of primary interest was the relative goodness-of-fit in the N400 time period (250-500 ms). As described in the introduction, this time period is known to reflect the linking of perceptual input to meaning. In previous work, both contextual support and related anomaly had their primary influence in this time period (Troyer and Kutas, 2020; see Figure 2 in the Results section, below). Notably, in previous work, effects of contextual support and related anomaly on the N400 were systematically influenced by the four individual differences measures examined here (Troyer and Kutas, 2018, 2020; Troyer and Federmeier, 2026; Troyer et al., 2022, 2026).

## Results

### Intercorrelations among measures of individual differences

Table 2 presents descriptive statistics of measures of individual differences. Figure 1 presents correlations between measures of individual differences (HP quiz scores, book-reading experience, movie-watching experience, and general reading experience). Notably, all four measures considered here are moderately-to-strongly correlated (Pearson's *r* between 0.39 and 0.66). It might seem surprising that HP book-reading and HP movie-watching were not as strongly correlated (at *r* = 0.39) as many of the other comparisons. However, this is a pattern we have seen in many of our studies, and is likely due in part to the fact that in general, almost all of the young adults in our studies had seen at least one Harry Potter movie, while many had never read a Harry Potter book; thus, there are likely many individuals who have engaged with the series in both modalities as well as many individuals who have only ever watched the movies.

### (r)ERP data

#### Grand-averaged ERP data

Figure 2 plots grand-averaged ERP by condition for a representative electrode (middle parietal; i.e., Pz in the 10–20 international system), as reported in Troyer and Kutas (2020). Differences scores are also visualized for each comparison of interest (contextual support effects, related anomaly effects). This figure also illustrates difference waves, demonstrating these effects are indeed dominant in the N400 time period analyzed here.

#### rERP estimates

**Figure 3:**
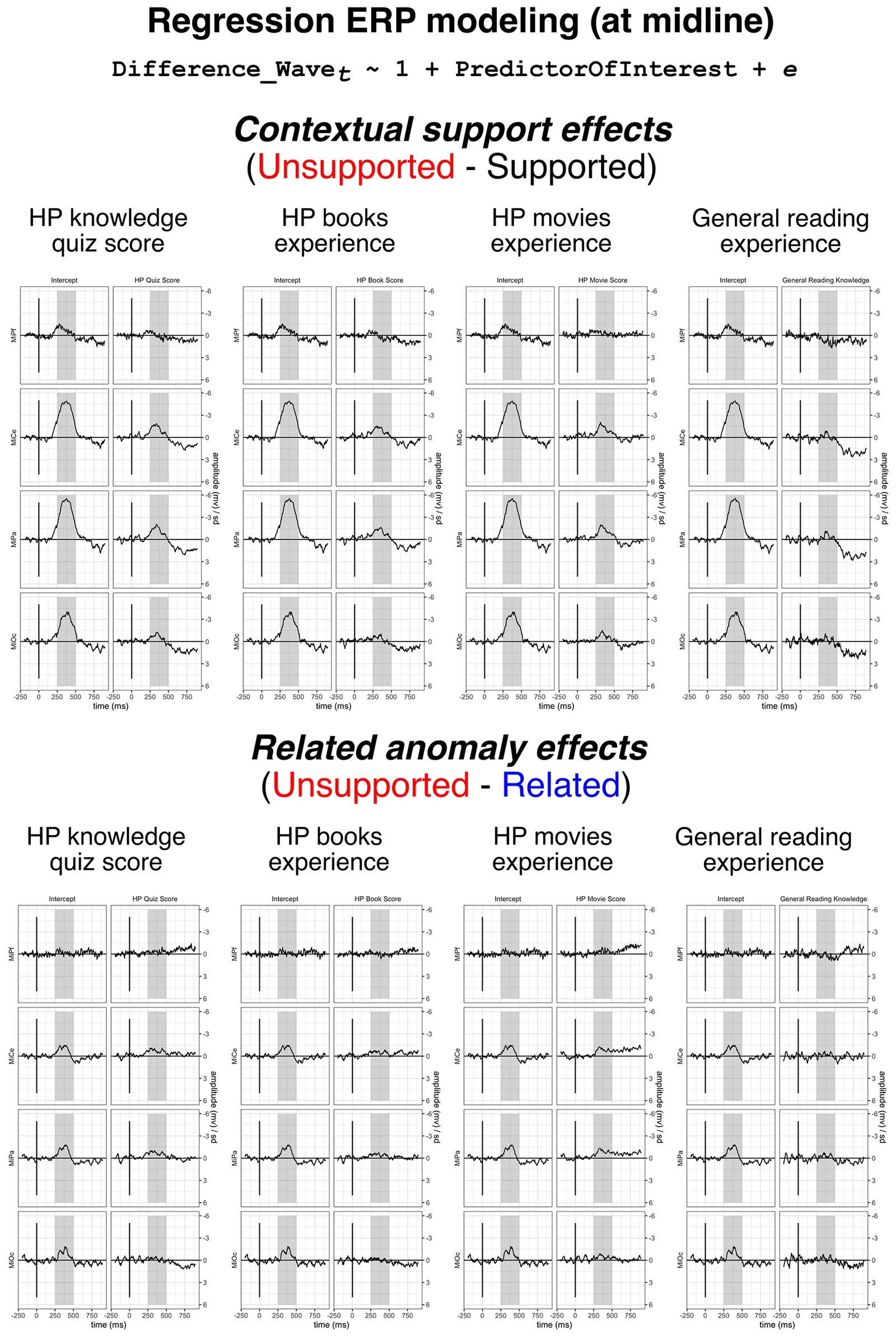
Each pair of panels shows the intercept term over time (i.e., the mean difference wave) next to the continuous effect of the predictors of interest for the contextual support effects **(left)** and related anomaly effects **(right)**. The highlighted regions correspond to the N400 time period (250–500 ms).

Figure 3 visualizes the rERP estimates for each effect (contextual support, related anomaly) and each model considered (simple linear regression models including an intercept term and as single individual difference measure as predictors). Estimates reflect beta coefficients from the linear regression models plotted at each time sample. For each model, the first coefficient reflects an intercept term. Given the modeling approach, this term is akin to a traditional ERP differences wave demonstrating categorical effects of condition. Thus, for the contextual support effects (unsupported-unrelated minus supported), the intercept rERP uses a different approach to show the same effect as in Figure 2 (difference waves). The second coefficient for each model reflects the individual differences term (HP knowledge score, HP book experience, HP movie experience, and general reading experience, respectively). These visualizations have *not* traditionally been possible without resorting to median splits that can notably reduce power (Humphreys, 1978). These visualizations go beyond traditional ERP difference waves in that they can directly estimate and visualize effects of continuous variables on an outcome variable (here, derived from a categorical effect of contextual support or related anomaly). Thus, each pair (intercept + individual difference measure) reflects the millisecond-by-millisecond estimates of weights that best fit the data when modeled with an intercept predictor and an individual difference predictor of interest (here, centered around zero via z-scoring).

#### AIC model comparison

**Figure 4:**
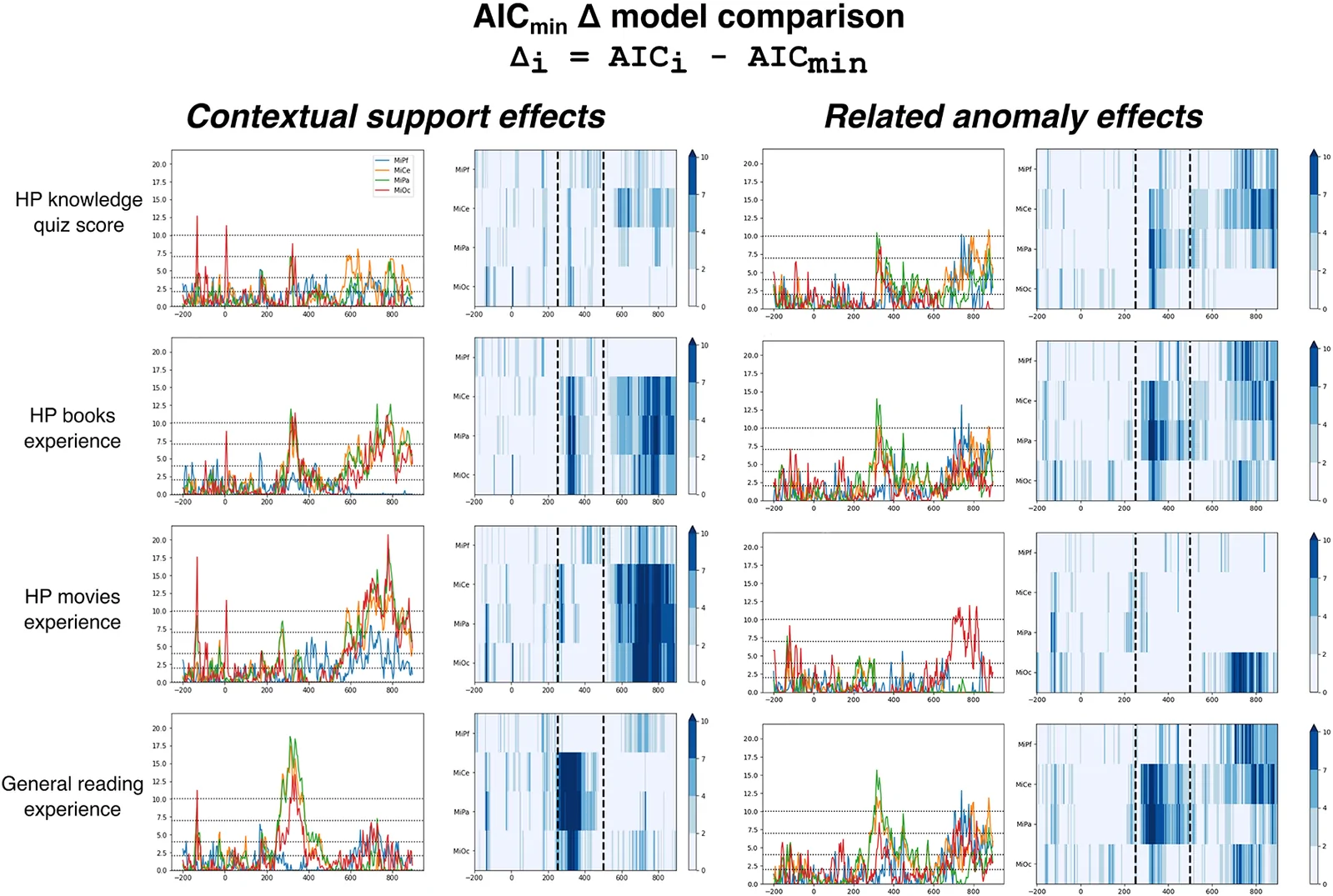
For each effect (left: contextual support; right: related anomaly), the left panel plots the raw AIC_min_ Δ for each regression model (over time) at four midline electrodes. Large values indicate that a model his minimal support for that time/electrode. The right panel plots these values discretely, with lighter colors corresponding to lower values (i.e., better fits) and darker colors corresponding to higher values. Based on the recommendations by Burnham and Anderson (2004), models with AIC_min_ Δ between 0 (i.e., the best-fitting model) and 2 receive substantial support; models with AIC_min_ Δ under 7 have some (but considerably less) support; and models above AIC_min_ Δ of 10 receive no support. By definition, one model always has the lowest AIC, meaning that that model's AIC_min_ Δ value is equal to 0 (at each electrode and time point).

**Figure 5:**
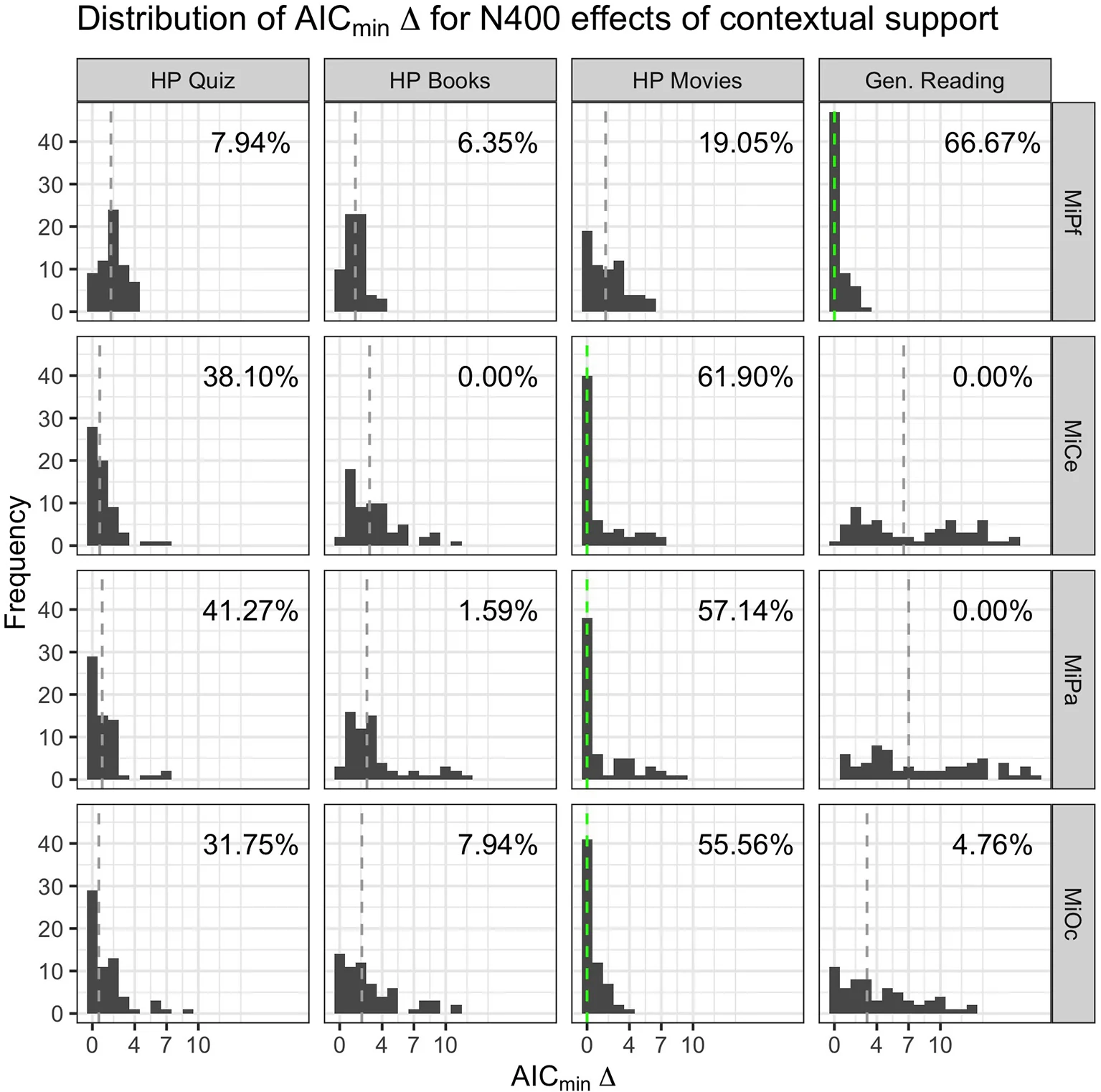
The distribution of AICmin Δ across the N400 time period (250–500 ms, sampled at 250 Hz) are plotted for each electrode and model for the effect of contextual support. Dashed lines indicate the median score for that electrode and model. Lines plotted in green indicate the model with the lowest median AIC_min_ Δ (by electrode). Percentages reflect the proportion of the time that that model, at that electrode, had the lowest AIC. HP movie experience outperformed all other measures for the central and parietal electrodes where N400 effects are most prominent.

**Figure 6:**
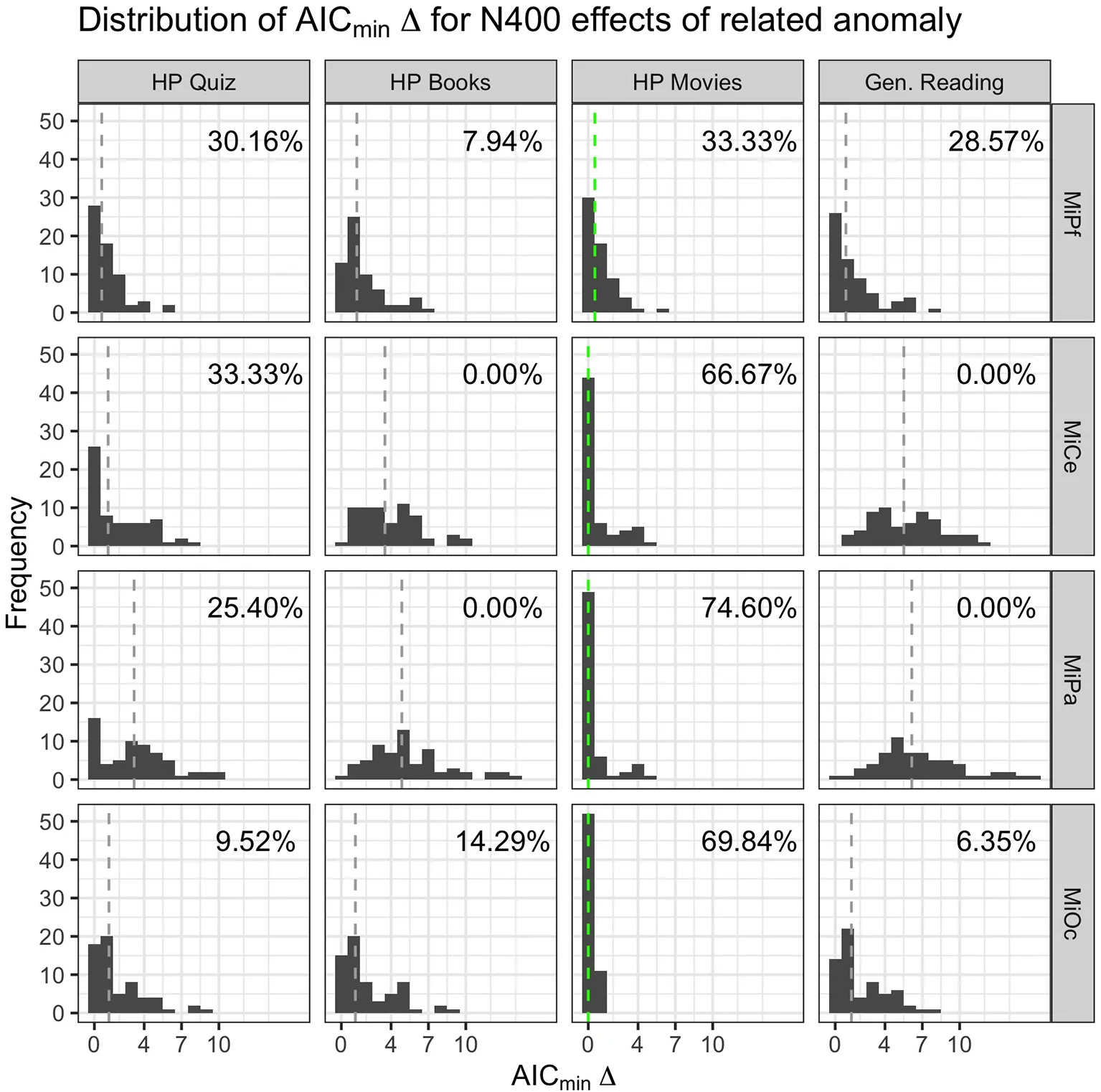
The distribution of AICmin Δ across the N400 time period (250–500 ms, sampled at 250 Hz) are plotted for each electrode and model for the effect of related anomaly. Dashed lines indicate the median score for that electrode and model. Lines plotted in green indicate the model with the lowest median AIC_min_ Δ (by electrode). Percentages reflect the proportion of the time that that model, at that electrode, had the lowest AIC. HP movie experience outperformed all other measures for the central and parietal electrodes where N400 effects are most prominent.

Figure 4 visualizes the AIC_min_ Δ metric for each effect of interest (contextual support, related anomaly), electrode (four midline electrodes from the front to the back of the head), and model (HP knowledge, HP reading experience, HP movie experience, and general reading experience). Plots on the left reflect the AIC_min_ Δ over time, with larger scores indicating poorer fit. Plots on the right use a binned approach to visualize these measures in bins of interest, with the lightest blue areas reflecting the lowest AIC_min_ Δ scores (0–2; i.e., the model has strong support) and the darkest blue areas reflecting the highest AIC_min_ Δ scores (7 and above; i.e., the model has no support). Broadly, to interpret these graphs: the lightest regions indicate that the model is the best fit, and darker regions indicate the model is a poor fit. The temporal N400 ROI (the area between 250 and 500 ms) is marked using dashed lines. For both effects (contextual support, related anomaly), neuroelectric activity in the N400 time period is generally well accounted for by the HP measures, which can visually be seen by the fact that majority of the AIC_min_ Δ scores for this time period are lower (often < 2, indicating no difference from the best fitting model) for the HP models compared to the model including general reading experience. This pattern is most apparent, for both effects considered, across the central and parietal channels where N400 effects are most prominent. In Table 3, we provide summary statistics showing that the measures of central tendency (mean and median) for AIC_min_ Δ values across the N400 time period in the central and parietal electrodes are indeed lower for the HP measures compared to the general reading experience measure. This is true for both the effects of contextual support and related anomaly. In Figures 5, 6, we further visualize the AIC_min_ Δ values across the N400 time period in histogram form, with the median values indicated for each electrode and model. These figures also illustrate that general reading experience is rarely the best-fitting model at any time point within the centro-parietal N400 effects considered here.

**Table 3:** Summary statistics (mean and median) for AIC_min_ Δ values, for each electrode and model, in the N400 time window (250–500 ms).

Measure	N400 effect of contextual support		
	Channel	Mean AIC_min_ Δ	Median AIC_min_ Δ
HP quiz	MiPf	1.90	1.75
	MiCe	1.02	0.70
	MiPa	1.13	0.93
	MiOc	1.38	0.61
HP books	MiPf	1.51	1.47
	MiCe	3.20	2.82
	MiPa	3.36	2.57
	MiOc	2.93	2.09
HP movies	MiPf	1.93	1.75
	MiCe	1.23	0.00
	MiPa	1.49	0.00
	MiOc	0.57	0.00
General reading	MiPf	0.38	0.00
	MiCe	7.48	6.54
	MiPa	8.38	7.01
	MiOc	4.33	3.08
	N400 effect of related anomaly		
HP quiz	MiPf	1.14	0.59
	MiCe	1.97	1.10
	MiPa	3.26	3.19
	MiOc	1.91	1.17
HP books	MiPf	1.85	1.25
	MiCe	3.97	3.50
	MiPa	5.40	4.86
	MiOc	2.11	1.13
HP movies	MiPf	0.93	0.53
	MiCe	0.78	0.00
	MiPa	0.57	0.00
	MiOc	0.18	0.00
General reading	MiPf	1.56	0.86
	MiCe	5.72	5.52
	MiPa	6.73	6.15
	MiOc	2.09	1.31

For each electrode, the model with the lowest median AIC_min_ Δ value is highlighted.

Notably, for both effects, there were also differences among the HP measures in terms of how well they explained the data. By directly comparing these different measures using the AIC_min_ Δ approach, it is clear that experience reading the HP books was *not* the best predictor; nor was the objectively derived measure from the HP trivia quizzes. Instead, it was the multimodal experience of movie watching that was the best predictor of both N400 effects. This is visually summarized in Figure 4 within the dashed boxes showing the N400 time window as well as in the highlighted boxes in Table 3. For the central and parietal electrodes where N400 activity is most prominent, the HP movie experience model has the lowest AIC (i.e., an AIC_min_ Δ value of 0) across the N400 time period the majority of the time (see exact percentages in Figures 5, 6). When compared to the HP movie experience measure, HP book experience tends to have an AIC_min_ Δ value >2, suggesting that the HP movie experience measure tends to have more explanatory power than book reading alone. This is especially true for the effects of related anomaly (see Table 3 for mean and median values for each electrode and model; see also median values plotted atop the histogram of AIC_min_ Δ values across the N400 time period in Figures 5, 6). By contrast, the measure of HP knowledge derived from the trivia quiz (while rarely outperforming the HP movie watching measure) consistently had an AIC_min_ Δ of < 2, suggesting its explanatory power was not distinguishable from that of the movie watching measure.

## Discussion

The present study compared how various measures of knowledge and experience related to millisecond-by-millisecond ERP time-course data recorded as individuals read sentences about a fictional world, one word at a time. As anticipated, in the N400 time period, all individual difference measures of HP knowledge and experience outperformed the general reading experience measure in explaining effects of contextual support and related anomaly. Perhaps surprisingly, within the HP measures of knowledge and experience, the multimodal experience of watching/listening to movies outperformed HP book experience (and, to a lesser extent, the objective measure of HP knowledge derived from a trivia quiz) in explaining these N400 effects.

These results lead to two important inferences. First, they confirm that, even though experience in the HP narrative/fictional domain is often correlated (and potentially conflated) with more general reading experience, the domain-specific measures considered out-performed general reading experience in explaining N400 effects of contextual support and related anomaly. This finding substantiates previous conclusions that domain-specific knowledge immediately impacts the available of contextually supported and contextually related knowledge from long-term (semantic) memory (Troyer and Kutas, 2018, 2020). Previous approaches using more traditional regression modeling had been unable to make this direct comparison given the collinearity of multiple individual differences measures.

The second important inference is that, while the domain of HP was chosen because of the rich fictional world created in a fantasy book series, it turns out that experience with the rich, multi-modal experience of watching movies was more predictive of linguistic effects during the reading comprehension experiment than book-reading experience. Note that this does *not* mean that the other HP measures (i.e., of HP book-reading or HP knowledge assessed via trivia quiz) are unpredictive of these N400 effects—rather, when pitted directly against one another, movie-watching experiences have the most explanatory power. The HP 7-book series includes, in total, approximately 4,100 pages (over a million words). The amount of time it would take to read this amount of text is likely to exceed 80 h, even for fast readers. By contrast, the 8 HP movies constitute only about 20 h of content, and spoken content is restricted to dialogue. Nonetheless, movie-watching experiences afford direct audiovisual experiences that are unavailable (at least in full form) to readers. Moreover, movie-watching experiences often take place in socially-situated contexts, such as movie theaters or living rooms. There may therefore be a wealth of contextual associations, including social ones, that are available during movie-watching but not during reading.

This latter finding provides evidence for how different types of experience can contribute to in-the-moment comprehension of linguistic input. This type of evidence can be difficult to come by, as it is difficult to empirically assess individuals' specific experiences. These findings highlight the importance of multimodal experience for language comprehension—in this case, from watching fictional movies. These findings are generally consistent with theories of grounded and embodied cognition proposing that we recruit perceptual processing mechanisms (i.e., the mechanisms involved in actually perceiving sensory input) in the service of bringing those same percepts to mind later, in the absence of the direct sensory (in this case, audiovisual) input.

Our findings also speak to the debate discussed in the introduction about the nature of linguistically-cued mental imagery vs. imagery that is perceptually available (e.g., via a picture or movie). Some theories have posited that the immersive experience of reading invokes a self-generated process of rich visual imagery that might be beneficial for subsequent memory—even extending to more general improvements in working memory and episodic memory (Stine-Morrow et al., 2022). The present study used a language comprehension task (not a direct memory task); however, given the important role of long-term, semantic memory in language comprehension, these findings suggest that, at least in some cases, perceptual/multimodal experiences may invoke relatively richer subsequent access to semantic long-term memory compared to purely linguistic experiences gleaned during reading.

Our findings also contribute to the growing body of literature investigating experience-based individual differences in language comprehension. To our knowledge, most studies of individual differences in language comprehension have focused either on generalized cognitive differences (e.g., in various executive functions, working memory, etc.; reviewed in Boudewyn, 2015) or variation in *language*-related experience—namely, print exposure/literacy (reviewed in Huettig and Hulstijn, 2024). One recent study provides a nuanced investigation into more specific knowledge gleaned from print exposure using a topicalized version of the author recognition task (the “tART”; Song et al., 2026). In that study, the authors used functional MRI to examine BOLD activity as individuals read fiction or non-fiction. Among individuals with relatively high print exposure, those with more diverse distributions of topical print exposure showed more diverse patterns of neural activity in regions of the default mode network, a brain network that is believed to reflect a “default” mode of processing supporting self-knowledge and introspection (Raichle et al., 2001; reviewed in Menon, 2023). They suggest that the DMN is particularly important for the “integrat[ion of] past experiences with ongoing processes of ‘sense-making”' (Song et al., 2026, p. 7). Notably, the relationship between topical print exposure diversity and DMN activity was limited to fiction reading and did not emerge during reading of expository, non-fiction text. The authors suggest that differences in specific topical knowledge may therefore underlie individual differences in how individuals relate narrative content, in particular, to their existing knowledge. In other words, when individuals have greater relevant existing knowledge to draw from, they may more readily link incoming language input with their own sense of self.

The engagement of the DMN during fiction reading is likely to be related to the notion of “absorption” (also termed “immersion” and “transportation”) during fiction reading, described as a “feeling of being in the story world” (Jacobs and Willems, 2018, p. 150) or being “gripped” by a story (Mak and Willems, 2019, p. 511). Indeed, the DMN is known to be strongly engaged when individuals read stories that require “mentalizing,” or imagining the state of mind of the characters (Tamir et al., 2016). In addition, higher levels of absorption (e.g., measured using the Story World Absorption Scale; Kuijpers et al., 2014) have been linked to greater engagement in perceptuomotor mental simulation, inferred from reading times for parts of passages that were scored high on perceptuomotor traits by a separate group of participants (Mak and Willems, 2019).

The present study may capture a similar phenomenon: individuals' specific background knowledge—here, measured only in a single fictional domain—predicts aspects of how they rapidly make use of sentence context to understand language about that same fictional domain, inferred from N400 brain potentials. Our results further suggest that knowledge gleaned from the multi-modal/audiovisual experience of movie-watching may in fact contribute to these sense-making processes to a larger extent than purely linguistic domain-related print exposure. It stands to reason that a greater availability of perceptuomotor simulation, gleaned from more perceptual experience during movie-watching, might better allow individuals to mentally simulate the events described in our short, two-story fictional descriptions. This mental simulation might have encouraged more direct engagement with the text, such that reader could become more immersed in the fictional world (i.e., more absorbed or transported by the text)—perhaps then facilitating not only access to the contextually appropriate critical words in our study but also to event-related content (even if linguistically anomalous).

It is important to acknowledge that this project was an exploratory re-analysis of existing data, and the original goals of the experiment did not include a direct examination of (nor a direct manipulation of) differences in the nature of experience driving ERP effects during language comprehension. Future work could experimentally manipulate the modality of input of fictional information, for example by assignment participants to read books vs. watch movies, and subsequently examine how the two experiences differentially shape language comprehension and memory processes.

In sum, these data show that experience with domain-specific content, in this case of a fictional world, outperform general measures of reading experience in explaining how people make meaning when understanding language (about that specific content). In addition, at least in some cases, the rich multimodal experiences of watching movies may relate relatively more strongly to knowledge availability during language understanding compared to purely linguistic experiences of reading. These findings run counter to theories on which meaning is based on linguistic experience alone, implicating multimodal contributions. This approach, comparing experience with a rich fictional world gleaned from reading vs. movie-watching, could be employed fruitfully to answer many questions about how different types of (multimodal) input shape knowledge organization and language comprehension.

## Data Availability

The data analyzed in this study are subject to the following licenses/restrictions: Data are unable because ERP data constitute biomedical signals and we are not permitted to share them under the institutional IRB/for ethical and privacy reasons. Requests to access these datasets should be directed to melissa.troyer@unlv.edu.

## Footnotes

1 Note: since it has previously been demonstrated that these measures explain N400 amplitudes above an intercept-only model (Troyer and Kutas, 2020), no intercept-only model was included in these comparisons; instead, models including measures of interest were directly compared.
